# Female Genito-Pelvic Pain/Penetration Disorder: Review of the Related Factors and Overall Approach

**DOI:** 10.1055/s-0038-1675805

**Published:** 2018-11-14

**Authors:** Ana Dias-Amaral, André Marques-Pinto

**Affiliations:** 1 Psychiatry and Mental Health Clinic, Centro Hospitalar de São João, Porto, Portugal; 2 Department of Urology, Centro Hospitalar do Porto, Porto, Portugal

**Keywords:** dyspareunia, vaginismus, vulvodynia, cognitive therapy, behavioral therapy, dispareunia, vaginismo, vulvodinia, terapia cognitiva, terapia comportamental

## Abstract

Genito-pelvic pain/penetration disorder (GPPPD) can be an extremely bothersome condition for patients, and a tough challenge for professionals regarding its assessment and treatment. The goal of the present paper is to review the etiology, assessment, and treatment of GPPPD, especially focusing on the cognitive aspects of the disease and cognitive-behavioral treatment options, through a non-systematic review of articles indexed to the Medline, Scopus and Web of Science databases, using the following MeSH queries: *pelvic pain; dyspareunia; vaginismus; vulvodynia;* and *cognitive therapy*. Altogether, 36 articles discussing the etiology, diagnosis and management of GPPPD were selected. We provide an overview of GPPPD based on biological, psychological and relational factors, emphasizing the last two. We also summarize the available medical treatments and provide strategies to approach the psychological trigger and persisting factors for the patient and the partner. Professionals should be familiarized with the factors underlining the problem, and should be able to provide helpful suggestions to guide the couple out of the GPPPD fear-avoidance circle.

## Resumo

A perturbação de dor gênito-pélvica e da penetração (PDGPP) é uma patologia com elevado impacto no bem-estar das pacientes, e traduz-se num desafio diagnóstico e de tratamento para os profissionais que as acompanham. O objetivo deste artigo é rever a etiologia e o tratamento da PDGPP, tendo em conta, principalmente, os aspetos cognitivos e as abordagens de inspiração psicoterapêutica cognitivo-comportamental. Para tal, foi efetuada uma revisão não sistemática dos artigos indexados às bases de dados Medline, Scopus e Web of Science, usando os termos: *dor pélvica*; *dispareunia*; *vaginismo*; *vulvodinia*; e *terapia cognitiva*. No total, foram incluídos 36 artigos discutindo a etiologia, diagnóstico e tratamento da PDGPP. Neste artigo, proporcionamos uma revisão do tratamento da PDGPP baseado em fatores biológicos, psicológicos e relacionais, enfatizando os últimos dois. Também resumimos as opções de tratamento médico disponíveis, e disponibilizamos estratégias para abordar os fatores desencadeantes e de manutenção tanto nas doentes quanto nos seus parceiros. Os profissionais devem estar familiarizados com os fatores subjacentes a este problema, e devem ser capazes de oferecer sugestões úteis para ajudar o casal a quebrar o círculo vicioso de medo e evitamento associado à PDGPP.

## Introduction

Until the publication of the fifth edition of the *Diagnostic and Statistical Manual of Mental Disorders* (DSM-5),^1^ women with pain associated to vaginal penetration were diagnosed either with dyspareunia or vaginismus, and dyspareunia was further categorized as either superficial (generalized or provoked vulvodynia) or deep.

It is important to consider that vaginismus may be secondary to dyspareunia;^2^ thus, the border between the two entities may be tenuous. Therefore, in the DSM-5 these entities were integrated in the same diagnostic category: genito-pelvic pain/penetration disorder (GPPPD). The diagnosis of GPPPD requires the presence of at least one of the following criteria:^1^ persistent or recurrent difficulties with vaginal penetration during intercourse; marked vulvovaginal or pelvic pain during vaginal intercourse or penetration attempts; marked fear or anxiety about vulvovaginal or pelvic pain in anticipation of, during, or as a result of vaginal penetration; or marked tensing or tightening of the pelvic floor muscles during attempted vaginal penetration. The additional criteria are similar to those of other sexual dysfunctions: presence of symptoms for at least six months, presence of significant distress, and symptoms not better explained by a diagnosis of non-sexual disturbance, causing significant relationship problems and not attributed to the effects of any substance or any other medical condition.

Around 14% to 34% of premenopausal women and ∼ 6.5% to 45% of postmenopausal women are affected by GPPPD.^3^ Occasional or transient pain appears to be four to eight times more frequent than chronic pain. In a Portuguese clinical population, the prevalence of vaginismus and dyspareunia was of 25.5% and 6.4% respectively.^4^ The presence of comorbidity is frequent. Almost half of the women with GPPPD also have another pain disorder, such as fibromyalgia, interstitial cystitis or irritable bowel syndrome.^5^ It is also associated with other sexual dysfunctions, such as female sexual interest/arousal disorder and low satisfaction with the sexual life.^3^

We hypothesize that this might be an underdiagnosed condition, possibly due to feelings of shame and hopelessness. Thus, we believe every physician dealing with women, their reproductive system and their sexual lives should be aware of the possible GPPPD causes and treatment options. Besides the biological factors and medical treatments, we believe every health professional dealing with GPPPD should be aware of the psychological factors that contribute to the persistence of the complaints and to hinder the therapeutic success. In the present paper, we aim to review the etiology, assessment, and treatment of GPPPD, especially focusing on the cognitive aspects of the disease and the cognitive-behavioral treatment options.

## Methods

We have performed a mini-review of systematic reviews and original articles regarding GPPPD diagnosis and treatment (and its former classification) indexed to the Medline, Scopus and Web of Science databases, and published between January 2000 and December 2017, using the following MeSH queries: *pelvic pain*; *dyspareunia*; *vaginismus*; vulvodynia; and *cognitive therapy*, which resulted in 53 articles. The inclusion criteria comprised current evidence regarding the biological factors that contribute to the etiology and the medical, surgical, and psychological treatments of GPPPD. A total of 7 papers were excluded, as they did not address GPPPD, but other causes of genital pain. Altogether, 36 articles discussing the etiology, diagnosis and treatment of GPPPD were deemed relevant by 2 separate reviewers, and were included in the final selection. Additionally, we have consulted one reference textbook^3^ because, to our knowledge, it is the most recently-updated published textbook on sex therapy.

## Results

The etiological factors can be divided into biological, psychological and relational, and they frequently coexist, highlighting the multifactorial nature of the conditions that cause genital pain.

Most of the conditions that cause genital pain are acute and transient, leading to skin and vulvar mucosa inflammation, usually due to infections – genital herpes or candidiasis, for example. Tissue lesions resulting from dermatological diseases (lichen planus, lichen sclerosus) also cause pain. Changes in the hormonal environment – and menopause is a classic example – can lead to vulvovaginal atrophy and consequent pain.^6^ Premalignant or malignant lesions of the vulva and their treatment, namely surgery and/or radiotherapy, can lead to anatomical, vascular and neurological changes, with consequences to neuronal pain pathways.^3^

Regarding the genetic factors, polymorphisms that cause increased vulnerability to inflammatory diseases were found in association with provoked vestibulodynia. Repeated urinary tract infections and early and prolonged use of oral contraceptives have also been associated with this condition.^3^ Some research suggests an increased pain sensitivity in these women, probably due to hyperinnervation, which may result from genetic, hormonal or inflammatory factors.^7^

Several studies have suggested an increase in resting muscle tone of the pelvic floor muscles in women with GPPPD, which may contribute to trigger and to the persistence of the complaints. This hypertonicity of the pelvic floor seems to be associated with decreased vaginal vasocongestion, with a possible contribution to deficient genital arousal, with consequent less lubrication and penetration pain.^8^ Table 1 summarizes the medical conditions that have been associated with GPPPD.^6^

**Table 1:** Medical causes of Genito-Pelvic Pain/Penetration Disorder

Superficial pain	Deep pain
Allergic reaction (e.g., latex)	Chronic pelvic pain syndrome
Congenital abnormalities (imperforate hymen, vaginal agenesis, vaginal septum)*	Crohn disease
Dermatologic diseases (lichen planus, lichen sclerosus)	Endometriosis
Fistula*	Hemorrhoids
Gynecological neoplasm*	Irritable bowel syndrome
Interstitial cystitis*	Neuropathies
Mechanical or chemical irritation	Pelvic floor muscle dysfunction
Pelvic organ prolapse*	Inflammatory pelvic disease
Perineal trauma*	Vaginitis
Pudendal nerve neuralgia*	
Infections (herpes, HPV, vaginosis)	
Atrophic vaginitis*	
Inadequate lubrication (arousal difficulties, estrogen deficit)*	

**Source:** Adapted from Bornstein et al.^6^

Note: *Conditions in which pain can be either superficial or deep.

The psychological factors are varied. Women with a GPPPD diagnosis are more likely to have a positive history of sexual, physical or emotional abuse.^3^ Pain complaints are also more frequent in women with history of depressive or anxious disorders.^9^ It has been hypothesized that there is a stress-induced central nervous system dysregulation that increases pain perception.^10^ It is important to acknowledge that the pelvic floor works as an emotional organ – anxiety causes reflex contractions of the pelvic muscles. A previous study shows that involuntary contractions of the pelvic diaphragm measured by electromyography in non-pathological women are more intense in states of anxiety than in response to a sexual threat.^8^ Increased pelvic floor tonus in response to threatening visual stimuli was also reported, suggesting that in these women vaginismus may be a conditioned protective response to penetration.^2^

Cognitive schemas, in the context of sexuality, are defined as nuclear ideas that individuals have about sexuality and about themselves as sexual beings. Individuals with sexual dysfunction show beliefs and expectations about sexuality that are usually unrealistic and inaccurate.^11^ Cognitive schemas have their origin in past experiences; they are reflected in current actions, and guide the future sexual behavior. Therefore, they may be predisposing factors to the development of sexual dysfunction. In women with sexual dysfunction, including vaginismus, there is a significantly greater activation of negative cognitive schemas, resulting in low affective involvement, avoidance of intimacy, and higher levels of anticipatory anxiety about abandonment.^12^ In these women, cognitive schemas of incompetence, difference/loneliness, self-deprecation^11^ ^12^ and rejection are frequently observed.^12^ In another study, women with sexual dysfunction, including vaginismus, had a significantly higher prevalence of early maladaptive schemas, namely in the impaired autonomy and performance domain: failure, dependence/incompetence, and vulnerability to danger were notorious.^13^ Overall, these results indicate that women with sexual dysfunction tend to interpret negative sexual events as a sign of personal incompetence and failure.

Negative cognitions about pain also seem to modulate its intensity. Hypervigilance refers to the permanent attention and monitoring of genital sensations that may signal the onset of pain. Catastrophization implies the inference of the worst possible consequence when pain is experienced. Both lead to an increase in pain experience and its possible negative consequences. Moreover, both associate positively with sexual dysfunction^9^ and predict a poor prognosis.^14^ Examples of negative beliefs related to pain are ‘pain is uncontrollable,’ ‘pain leads to disability,’ and ‘all activity should be avoided.’^5^ Dysfunctional beliefs related to age (‘in women sexual desire decreases with increasing age’) were also common among women with vaginismus.^12^ On the other hand, fewer negative cognitions related to pain and more positive cognitions related to penetration are associated with higher couple satisfaction and better sexual function.^14^

Automatic thoughts were conceptualized by Aaron Beck as images or cognitions that result from the activation of cognitive schemes at particular moments.^15^ Thus, these thoughts or images reflect the meaning that the individual attributes to a given situation. In a population of women with sexual dysfunction, including GPPPD, a significantly higher prevalence of thoughts of sexual abuse and failure/disengagement and absence of erotic thoughts was noticed.^12^ ^15^ The emotional response to these thoughts also seems to vary between women with and without sexual dysfunction: women with sexual dysfunction, including GPPPD, mention more often sadness, guilt, disappointment and anger, whereas women without dysfunction mention sexual pleasure and satisfaction.^16^ Examples of automatic thoughts are ‘penetration is impossible’ or ‘it will always cause pain, and this pain will be unbearable.’^17^

**Fig. 1:**
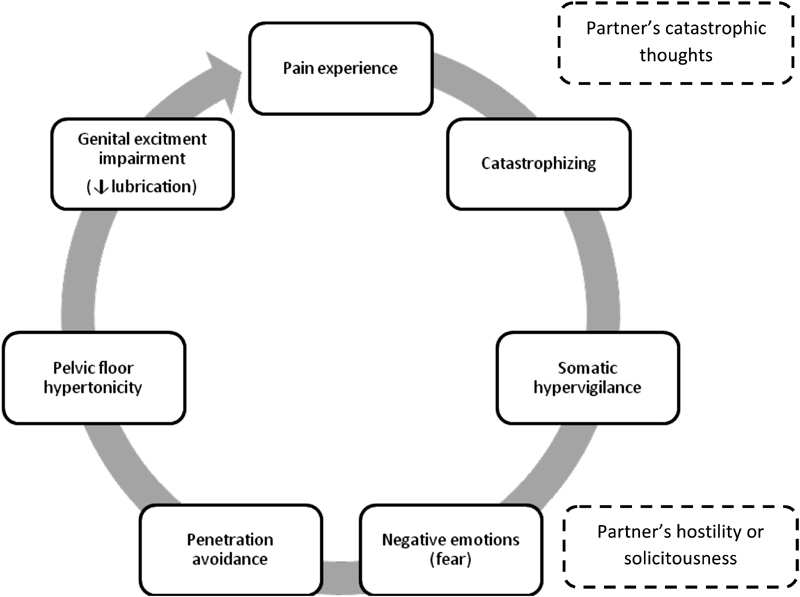
The vicious circle of female genital pain. **Source:** Adapted from Basson.^10^

Although there has been some research in this field in recent years, it is not yet possible to understand whether the psychological differences between women with or without genital pain are a cause or a consequence. They seem to play a role as predisposing and persisting factors, as they are essential in establishing positive coping and pain-reduction strategies. The development and persistence of GPPPD has been conceptualized as a vicious circle.^10^ The fear-avoidance model of chronic pain has been used to explain the persistence of pain in GPPPD (Fig. 1). An initial painful experience produces fearful and catastrophic thoughts about pain and its meaning. These lead to somatic hypervigilance that amplifies all potentially negative sensations, increasing the negative emotions associated with pain and the avoidance of sexual activity.^10^ ^14^ Pelvic floor hypertonicity secondarily exacerbates this experience. Pain impairs genital excitement, leading to less lubrication and painful penetration. Repeated experiences of sexual pain confirm fear and the need for vigilance,^17^ contributing to vaginal penetration avoidance.^10^ At last, the avoidance of sexual activity prevents automatic thoughts from being disconfirmed.^17^

Although pain is experienced by the woman, it is important to acknowledge that it also affects the partner. The fear of pain leads to penetration avoidance and ultimately to partner avoidance.^6^ Sexual communication between the couple improves the sexual satisfaction of women, possibly by enabling an open discussion about pain and by increasing the couple's sexual repertoire.^9^ ^14^

Conversely, partner response to female pain seems to influence her perception: men who encourage adaptive coping strategies and reinforce attempts to have partnered sex are associated with lower pain rates in women and improved overall sexual functioning. On the other hand, both hostile men, who are easily enraged by any sign of pain, and overly understanding and solicitous men, who immediately stop all sexual activity at the first sign of discomfort of the partner, are associated with increased pain, more depressive symptoms, and lower marital satisfaction. The explanation seems to be that an overly sympathetic partner does not stimulate the search for adaptive responses to pain, but rather the avoidance of sexual intercourse.^18^ Differences among partner responses seem to be associated with their own cognitive distortions.^19^ The negative pain attributions (internality – personal responsibility; globality – the problem affects all dimensions of life; and stability – persistence of the problem in the future) made by the partner increase their distress.^20^

### Genito-Pelvic Pain/Penetration Disorder (GPPPD) Assessment

Genito-pelvic pain/penetration disorder is difficult to diagnose and to treat, so it can become a frustrating condition for both the patient and the therapist.^5^ When a woman complains about genital pain, an exhaustive evaluation is necessary to establish a probable etiology, whenever possible. A first evaluation implies a general medical evaluation: characterization of the complaint (acquired or lifelong, situational or generalized, provoked or spontaneous) and investigation of the medical, surgical, gynecological, sexual, psychiatric, and drug histories. Genito-pelvic examination is mandatory, including “pain mapping,” vaginal pH measurement, and evaluation of the pelvic floor tonus. According to the history and physical examination findings, complementary exams such as biopsies or ultrasounds may be required.^21^

### Genito-Pelvic Pain/Penetration Disorder (GPPPD) Treatment

After establishing a probable etiology, the therapeutic approach should be defined by a multidisciplinary team.^22^ The best strategy usually results from the combination of several therapeutic modalities. There is evidence of efficacy for both the medical therapy and the surgery. Topical applications of anesthetics and corticosteroids appear to moderately decrease pain in dyspareunia. Some studies have reported that these treatments are more effective in generalized vulvodynia, as the pain is constant and unprovoked.^3^ In provoked vestibulodynia, cognitive-behavioral therapy appears to be superior to corticosteroids in reducing pain catastrophization.^23^ Actually, the presence of catastrophic thoughts, fear and avoidance predict a poor response to the medical therapy.^24^ On the other hand, feelings of self-efficacy predict a positive response.^24^ Low doses of tricyclic antidepressants and anticonvulsants, such as in other chronic pain disorders, are also a popular treatment in vulvodynia, but current evidence seems to contraindicate their use.^21^ Topical anesthetics and corticosteroids and injections of botulinum toxin have been tested in the treatment of vaginismus, but the evidence for their benefit is modest, and they are not recommended as a first-line treatment.^21^

Surgery (vestibulectomy) in cases of localized vulvodynia is effective when other options fail. This procedure involves the excision of the vestibular area where the pain originates. Overall, the success rate of the surgery regarding pain management appears to be twice as high as cognitive-behavioral psychotherapy and electromyographic biofeedback,^25^ with success rates of 60% to 90%.^21^ The treatment benefits endure at least two and a half years, and are predicted by pretreatment pain intensity and the presence of fear/avoidance schemas.^25^ However, despite contributing to pain control, surgery may decrease the vulvar region's sensitivity to pleasure, which may ultimately worsen the overall sexual satisfaction.^6^ On the other hand, a combination approach of physical therapy and psychosexual therapy in provoked vestibulodynia seems as effective as surgery.^26^

Electromyographic biofeedback also appears to have some therapeutic success. It consists of the insertion in the vagina of an electromyography sensor that provides the woman with information about the degree of muscle contraction of the pelvic floor.^21^ Relaxation exercises are then performed. The association of this modality with the cognitive-behavioral intervention may increase the efficacy of the treatment in cases of vaginismus.^27^ Pelvic floor physiotherapy and electrostimulation seem to have benefits too, both in dyspareunia and vaginismus.^26^ The goals of these interventions are to decrease the degree of muscle tension at rest, increase the attention directed to this muscle group and its control, increase the elasticity of the vaginal introitus, and expose the patients to penetration.^28^ The latter situation may be especially beneficial in women with high levels of fear and anxiety related to penetration, as it provides a calm and secure environment for a more gradual and comfortable contact with penetration.^6^

When a specific etiological diagnosis is made, treatment should be directed to the primary condition. Cognitive-behavioral psychotherapy has been the most popular and studied psychotherapeutic intervention in GPPPD, and can be performed individually, as a couple or as a group.^29^ The main targets of the therapy are cognitive distortions, emotional dysregulation, and maladaptive behaviors that perpetuate symptoms and disturb the couple's relationship.^9^ This kind of treatment is useful in cases in which psychological and/or relational issues are the predominant components, and several studies have proven its efficacy.^2^ ^17^ ^22^ ^23^ ^28^ ^29^ ^30^ ^31^ ^32^ ^33^ ^34^ ^35^ The choice of the most appropriate intervention should be based on the assessment of the various dimensions of pain (Table 2).^6^

**Table 2:** Dimensions of pain

	Characteristics
Physiological	Etiology and duration of pain
Sensorial	Location, intensity and quality of pain
Affective	Emotional response to pain (anxiety, sadness, fear, despair, concern etc.)
Cognitive	Underlying thoughts, meaning of pain and degree of focus on it, coping strategies used, attitudes and beliefs
Behavioral	Pain indicators, behaviors that aim to control pain, how the patient communicates the presence of pain and associated symptoms
Sociocultural	Sociodemographic variables, cultural context (lack of sexual knowledge, internalized negative messages in relation to sexuality), social roles played (in the family, in the workplace), and family history

**Source:** Adapted from Bornstein et al.^6^

Another key point is the establishment of realistic therapeutic goals. For many women, therapeutic success is defined as total elimination of pain, but some fail to achieve this goal. Some examples of positive treatment outcomes^31^ in both dyspareunia and vaginismus are reduction of pain from severe to moderate or mild; reduction of muscular tension in the perineum/pelvis; reduction of negative cognitions related to pain (less frequent catastrophic thoughts and the ability to assess pain-generating situations in a more positive way); positive coping (the ability to focus on the positive components of sexual experience); and improvement in sexual functioning (exploration of expressions of sexuality that do not include intercourse, and the ability to communicate their own needs to the partner).

The initial therapeutic approach must be psychoeducation of the couple.^36^ Neither the patient nor the partner should face this stage performing a passive role: it is an opportunity to understand the problem, to learn about female anatomy, and to challenge myths. The couple should also be informed about the biopsychosocial nature of GPPPD and the role of psychological and marital issues as triggers and persistence factors.^5^ The couple should be provided with behavioral strategies that can improve pain (Table 3).

**Table 3:** Behavioral strategies to reduce pain

Situation	Behavioral strategies
Intercourse	Use water-based lubricants Explore different coital positions Sitz bath or ice packs after intercourse Urinate immediately after intercourse
Physical activity	Avoid exercises that put pressure on the vulvar region or that produce friction (e.g., cycling) Avoid swimming pools or saunas/steam baths
Personal hygiene	Avoid scented products Do not wash the genital region more than once a day Do not shave the vulvar region
Clothing	Avoid tight underwear or trousers Remove wet clothing immediately after an activity Use cotton underwear (avoid synthetic fabrics) Wash your clothes with hypoallergenic products

**Source:** Adapted from Bornstein et al.^6^

Another important goal in the initial approach to GPPPD should be anxiety reduction. It is not uncommon that, when presenting to the therapist, the couple is stuck in an avoidance circle: avoidance of intimacy, of problem discussion, of search for solutions and, ultimately, of sexual activity. When they finally look for treatment, they are likely to feel anxious because it will be necessary to discuss the problem and eventually resume what they have been actively avoiding: sex. It is important that the therapist is aware of this situation and positively reinforces the fact that the woman or the couple has sought help. It is crucial to inform them that the therapy will focus on increasing the couple's desire, excitement and intimacy, and not on increasing the frequency of penetrative sex. Intercourse is not a primary goal, but a consequence of a successful therapy.^3^

In a second stage of the treatment, it is important that the therapist challenges certain cognitions about sex that are common among couples. Two common cognitive distortions in these women are hypervigilance and catastrophic pain. Challenging these distortions is essential to lessen emotional reactivity. On the other hand, the use of sexual fantasies should be encouraged, since positive sexual cognitions increase desire and arousal, which can increase lubrication and pleasure, and reduce pain.

The couple should also be encouraged to actively express their emotions and to display physical affection. The goal is to uncouple physical affection and anticipation of genital pain, that is, to reduce anticipatory anxiety. This can be achieved through the sensate focus technique developed by Masters and Johnson and published in 1970 in their book Human Sexual Inadequacy.^37^ The goal is to move from non-genital touch to genital touch and finally to penetration. At the beginning, penetration is forbidden, which usually reduces the patient's anxiety, allowing her to focus on pleasant bodily sensations. This gradual exposure to physical contact usually results in increased desire and arousal and reduced pain. Sensate focus is also useful in expanding the couple's sexual repertoire. Increased control over pain seems to mediate the efficacy of these interventions.^35^

In the particular case of vaginismus, and because muscle contraction is considered a conditioned response to fear, exposure methods are usually preferred.^2^ The use of progressively larger vaginal dilators (systematic desensitization) associated with a physiotherapy program should be strongly encouraged.^36^ The efficacy of this treatment appears to be mediated by avoidance behavior^2^ and reduction of cognitive distortions, and by the increased control over pain.^34^ By the end of a cognitive-behavioral program, women's anxiety levels decrease, and marital harmony and global sexual satisfaction improve.^38^

## Final Considerations

Although the origins of GPPPD are not always evident, cognitions, emotions and behaviors that perpetuate the complaints are certainly identifiable. We believe most couples can overcome these issues and engage in a more satisfying sex life without the need of intensive sex therapy. In order for this to happen, family physicians and gynecologists should be familiarized with the factors underlining the problem, and should be able to provide helpful suggestions to guide the couple out of the GPPPD fear-avoidance circle. Helping the patient and partner identify the triad of factors that contribute to the persistence of GPPPD (cognitions, emotions and behaviors associated to pain) can improve the symptoms, assist in the adaptation to them, and prevent their resurgence.

## References

[1] American Psychiatric Association. Diagnostic and Statistical Manual of Mental Disorders (DSM-5). 5th ed Washington, DC APA 2013

[2] ter Kuile M M van Lankveld J J de Groot E Melles R Neffs J Zandbergen M Cognitive-behavioral therapy for women with lifelong vaginismus: process and prognostic factors Behav Res Ther 2007 45 02 359 373 Doi: 10.1016/j.brat.2006.03.013 16701078 10.1016/j.brat.2006.03.013

[3] Meana M Fertel E Maykut C Treating genital pain associated with sexual intercourse Chichester Wiley Blackwell 2017 98 114

[4] Nobre P J Pinto-Gouveia J Gomes F A Prevalence and comorbidity of sexual dysfunctions in a Portuguese clinical sample J Sex Marital Ther 2006 32 02 173 182 Doi: 10.1080/00926230500442334 16418107 10.1080/00926230500442334

[5] Weijenborg P T Ter Kuile M M Stones W A cognitive behavioural based assessment of women with chronic pelvic pain J Psychosom Obstet Gynaecol 2009 30 04 262 268 Doi: 10.3109/01674820903378742 19922399 10.3109/01674820903378742

[6] Bornstein J Goldstein A T Stockdale C K 2015 ISSVD, ISSWSH, and IPPS Consensus terminology and classification of persistent vulvar pain and vulvodynia J Sex Med 2016 13 04 607 612 Doi: 10.1016/j.jsxm.2016.02.167 27045260 10.1016/j.jsxm.2016.02.167

[7] Pukall C F Goldstein A T Bergeron S Vulvodynia: definition, prevalence, impact, and pathophysiological factors J Sex Med 2016 13 03 291 304 Doi: 10.1016/j.jsxm.2015.12.021 26944461 10.1016/j.jsxm.2015.12.021

[8] Both S van Lunsen R Weijenborg P Laan E A new device for simultaneous measurement of pelvic floor muscle activity and vaginal blood flow: a test in a nonclinical sample J Sex Med 2012 9 11 2888 2902 Doi: 10.1111/j.1743-6109.2012.02910.x 22925559 10.1111/j.1743-6109.2012.02910.x

[9] Bergeron S Likes W M Steben M Psychosexual aspects of vulvovaginal pain Best Pract Res Clin Obstet Gynaecol 2014 28 07 991 999 Doi: 10.1016/j.bpobgyn.2014.07.007 25104563 10.1016/j.bpobgyn.2014.07.007

[10] Basson R The recurrent pain and sexual sequelae of provoked vestibulodynia: a perpetuating cycle J Sex Med 2012 9 08 2077 2092 Doi: 10.1111/j.1743-6109.2012.02803.x 22672388 10.1111/j.1743-6109.2012.02803.x

[11] Nobre P J Pinto-Gouveia J Cognitive schemas associated with negative sexual events: a comparison of men and women with and without sexual dysfunction Arch Sex Behav 2009 38 05 842 851 Doi: 10.1007/s10508-008-9450-x 19191018 10.1007/s10508-008-9450-x

[12] Nobre P J Pinto-Gouveia J Cognitive and emotional predictors of female sexual dysfunctions: preliminary findings J Sex Marital Ther 2008 34 04 325 342 Doi: 10.1080/00926230802096358 18576234 10.1080/00926230802096358

[13] Oliveira C Nobre P J Cognitive structures in women with sexual dysfunction: the role of early maladaptive schemas J Sex Med 2013 10 07 1755 1763 Doi: 10.1111/j.1743-6109.2012.02737.x 22524501 10.1111/j.1743-6109.2012.02737.x

[14] Anderson A B Rosen N O Price L Bergeron S Associations between penetration cognitions, genital pain, and sexual well-being in women with provoked vestibulodynia J Sex Med 2016 13 03 444 452 Doi: 10.1016/j.jsxm.2015.12.024 26853045 10.1016/j.jsxm.2015.12.024

[15] Nobre P J Pinto-Gouveia J Differences in automatic thoughts presented during sexual activity between sexually functional and dysfunctional men and women Cognit Ther Res 2008 32 37 49 Doi: 10.1007/s10608-007-9165-7

[16] Nobre P J Pinto-Gouveia J Emotions during sexual activity: differences between sexually functional and dysfunctional men and women Arch Sex Behav 2006 35 04 491 499 Doi: 10.1007/s10508-006-9047-1 16909318 10.1007/s10508-006-9047-1

[17] van Lankveld J J ter Kuile M M de Groot H E Melles R Nefs J Zandbergen M Cognitive-behavioral therapy for women with lifelong vaginismus: a randomized waiting-list controlled trial of efficacy J Consult Clin Psychol 2006 74 01 168 178 16551154 10.1037/0022-006X.74.1.168

[18] Rosen N O Bergeron S Sadikaj G Glowacka M Delisle I Baxter M L Impact of male partner responses on sexual function in women with vulvodynia and their partners: a dyadic daily experience study Health Psychol 2014 33 08 823 831 Doi: 10.1037/a0034550 24245835 10.1037/a0034550

[19] Davis S N Bergeron S Sadikaj G Corsini-Munt S Steben M Partner behavioral responses to pain mediate the relationship between partner pain cognitions and pain outcomes in women with provoked vestibulodynia J Pain 2015 16 06 549 557 Doi: 10.1016/j.jpain.2015.03.002 25827063 10.1016/j.jpain.2015.03.002

[20] Jodoin M Bergeron S Khalifé S Dupuis M J Desrochers G Leclerc B Male partners of women with provoked vestibulodynia: attributions for pain and their implications for dyadic adjustment, sexual satisfaction, and psychological distress J Sex Med 2008 5 12 2862 2870 Doi: 10.1111/j.1743-6109.2008.00950.x 18637992 10.1111/j.1743-6109.2008.00950.x

[21] Goldstein A T Pukall C F Brown C Bergeron S Stein A Kellogg-Spadt S Vulvodynia: Assessment and Treatment J Sex Med 2016 13 04 572 590 Doi: 10.1016/j.jsxm.2016.01.020 27045258 10.1016/j.jsxm.2016.01.020

[22] Breton A Miller C M Fisher K Enhancing the sexual function of women living with chronic pain: a cognitive-behavioural treatment group Pain Res Manag 2008 13 03 219 224 18592058 10.1155/2008/369382 PMC2671310

[23] Bergeron S Khalifé S Dupuis M J McDuff P A randomized clinical trial comparing group cognitive-behavioral therapy and a topical steroid for women with dyspareunia J Consult Clin Psychol 2016 84 03 259 268 Doi: 10.1037/ccp0000072 26727408 10.1037/ccp0000072

[24] Desrochers G Bergeron S Khalifé S Dupuis M J Jodoin M Provoked vestibulodynia: psychological predictors of topical and cognitive-behavioral treatment outcome Behav Res Ther 2010 48 02 106 115 Doi: 10.1016/j.brat.2009.09.014 19879555 10.1016/j.brat.2009.09.014

[25] Bergeron S Khalifé S Glazer H I Binik Y M Surgical and behavioral treatments for vestibulodynia: two-and-one-half year follow-up and predictors of outcome Obstet Gynecol 2008 111 01 159 166 Doi: 10.1097/01.AOG.0000295864.76032.a7 18165405 10.1097/01.AOG.0000295864.76032.a7

[26] Backman H Widenbrant M Bohm-Starke N Dahlof L G Combined physical and psychosexual therapy for provoked vestibulodynia-an evaluation of a multidisciplinary treatment model J Sex Res 2008 45 04 378 385 Doi: 10.1080/00224490802398365 18937129 10.1080/00224490802398365

[27] Seo J T Choe J H Lee W S Kim K H Efficacy of functional electrical stimulation-biofeedback with sexual cognitive-behavioral therapy as treatment of vaginismus Urology 2005 66 01 77 81 Doi: 10.1016/j.urology.2005.01.025 15992873 10.1016/j.urology.2005.01.025

[28] Brotto L A Yong P Smith K B Sadownik L A Impact of a multidisciplinary vulvodynia program on sexual functioning and dyspareunia J Sex Med 2015 12 01 238 247 Doi: 10.1111/jsm.12718 25354520 10.1111/jsm.12718

[29] Goldfinger C Pukall C F Thibault-Gagnon S McLean L Chamberlain S Effectiveness of cognitive-behavioral therapy and physical therapy for provoked vestibulodynia: a randomized pilot study J Sex Med 2016 13 01 88 94 Doi: 10.1016/j.jsxm.2015.12.003 26755091 10.1016/j.jsxm.2015.12.003

[30] Bergeron S Binik Y M Khalifé S A randomized comparison of group cognitive--behavioral therapy, surface electromyographic biofeedback, and vestibulectomy in the treatment of dyspareunia resulting from vulvar vestibulitis Pain 2001 91 03 297 306 Doi: 10.1016/S0304-3959(00)00449-8 11275387 10.1016/S0304-3959(00)00449-8

[31] Engman M Wijma K Wijma B Long-term coital behaviour in women treated with cognitive behaviour therapy for superficial coital pain and vaginismus Cogn Behav Ther 2010 39 03 193 202 Doi: 10.1080/16506070903571014 20390584 10.1080/16506070903571014

[32] Lofrisco B M Female sexual pain disorders and cognitive behavioral therapy J Sex Res 2011 48 06 573 579 Doi: 10.1080/00224499.2010.540682 21184338 10.1080/00224499.2010.540682

[33] Lindström S Kvist L J Treatment of Provoked Vulvodynia in a Swedish cohort using desensitization exercises and cognitive behavioral therapy BMC Womens Health 2015 15 108 Doi: 10.1186/s12905-015-0265-3 26603697 10.1186/s12905-015-0265-3 PMC4659238

[34] Ter Kuile M M Melles R J Tuijnman-Raasveld C C de Groot H E van Lankveld J J Therapist-aided exposure for women with lifelong vaginismus: mediators of treatment outcome: a randomized waiting list control trial J Sex Med 2015 12 08 1807 1819 Doi: 10.1111/jsm.12935 26247327 10.1111/jsm.12935

[35] ter Kuile M M Weijenborg P T A cognitive-behavioral group program for women with vulvar vestibulitis syndrome (VVS): factors associated with treatment success J Sex Marital Ther 2006 32 03 199 213 Doi: 10.1080/00926230600575306 16809249 10.1080/00926230600575306

[36] Dunkley C R Brotto L A Psychological treatments for provoked vestibulodynia: integration of mindfulness-based and cognitive behavioral therapies J Clin Psychol 2016 72 07 637 650 Doi: 10.1002/jclp.22286 27019368 10.1002/jclp.22286

[37] Masters WH Johnson VE. Human Sexual Inadequacy Boston: Little, Brown 1970

[38] Kabakçi E Batur S Who benefits from cognitive behavioral therapy for vaginismus? J Sex Marital Ther 2003 29 04 277 288 Doi: 10.1080/00926230390195515 14504016 10.1080/00926230390195515

